# Infant feeding knowledge, perceptions and practices among women with and without HIV in Johannesburg, South Africa: a survey in healthcare facilities

**DOI:** 10.1186/s13006-017-0109-x

**Published:** 2017-04-08

**Authors:** Coceka N. Mnyani, Carol L. Tait, Jean Armstrong, Duane Blaauw, Matthew F. Chersich, Eckhart J Buchmann, Remco P. H. Peters, James A McIntyre

**Affiliations:** 1 Department of Obstetrics and Gynaecology, School of Clinical Medicine, University of the Witwatersrand, 7 York Road, Parktown, 2193 Johannesburg, South Africa; 2 School of Public Health, University of the Witwatersrand, Johannesburg, South Africa; 3 SACEMA (DST/NRF Centre of Excellence in Epidemiological Modelling and Analysis), Stellenbosch University, Stellenbosch, South Africa; 4 Anova Health Institute, Johannesburg, South Africa; 5 HIVSA, Johannesburg, South Africa; 6 Wits Reproductive Health and HIV Institute, Faculty of Health Sciences, University of the Witwatersrand, Johannesburg, South Africa; 7 International Centre for Reproductive Health, Department of Obstetrics and Gynaecology, University of Ghent, Ghent, Belgium; 8 School of Public Health and Family Medicine, University of Cape Town, Cape Town, South Africa

**Keywords:** Breastfeeding, Infant feeding, HIV, PMTCT, South Africa

## Abstract

**Background:**

South Africa has a history of low breastfeeding rates among women with and without Human Immunodeficiency Virus (HIV). In this study, we assessed infant feeding knowledge, perceptions and practices among pregnant and postpartum women with and without HIV, in the context of changes in infant feeding and Prevention of Mother-to-Child Transmission of HIV (PMTCT) guidelines.

**Methods:**

This was a cross-sectional survey conducted from April 2014 to March 2015 in 10 healthcare facilities in Johannesburg, South Africa. A total of 190 pregnant and 180 postpartum women (74 and 67, respectively, were HIV positive) were interviewed using a semi-structured questionnaire. Multiple regression analyses assessed factors associated with an intention to exclusively breastfeed, and exclusive breastfeeding of infants less than six months of age.

**Results:**

Women with HIV had better overall knowledge on safe infant feeding practices, both in general and in the context of HIV infection. There were however gaps in knowledge among women with and without HIV. Information from healthcare facilities was the main source of information for all groups of women in the study. A greater percentage of women without HIV 80.9% (93/115), reported an intention to exclusively breastfeed, compared to 64.9% (48/74) of women with HIV, *p* = 0.014. Not having HIV was positively associated with a reported intention to breastfeed, Adjusted Odds Ratio (AOR) 3.60, 95% CI 1.50, 8.62. Other factors associated with a reported intention to exclusively breastfeed were prior breastfeeding experience and higher knowledge scores on safe infant feeding practices in the context of HIV infection. Among postpartum women, higher scores on general knowledge of safe infant feeding practices were positively associated with reported exclusive breastfeeding, AOR 2.18, 95% CI 1.52, 3.12. Most women perceived that it was difficult to exclusively breastfeed and that cultural factors were a barrier to exclusive breastfeeding.

**Conclusions:**

While a greater proportion of women are electing to breastfeed, HIV infection and cultural factors remain an important influence on safe infant feeding practices. Healthcare workers are the main source of information, and highlight the need for accurate and consistent messaging for both women with and without HIV.

## Background

Safe infant feeding practices remain an integral part of prevention of mother-to-child transmission of HIV (PMTCT). The 2010 World Health Organization (WHO) guidelines on infant feeding in the context of HIV infection recommend that infant feeding practices should support the greatest likelihood of infant HIV-free survival, while also protecting against non-HIV morbidity and mortality [1]. Countries were encouraged to support one infant feeding option; either breastfeeding with antiretroviral (ARV) interventions, or avoidance of all breastfeeding by women with HIV [1]. Up until 2011, South Africa supported both options, with the provision of a free six months’ supply of infant formula for women with HIV who elected to formula feed. Amidst much criticism about supporting two options and the likelihood of confusion and inappropriate feeding practices, South Africa then selected one strategy; that of promoting exclusive breastfeeding for women with HIV, and thus withdrew the provision of free infant formula [2–4]. The current recommendation is for women with HIV to breastfeed for up to 12 months, with provision of infant and maternal ARVs [5].

South Africa has a history of low breastfeeding rates among both women with and without HIV [6]. While breastfeeding initiation rates at birth are high, very few women exclusively breastfeed for six months, with cultural factors one of the main drivers of mixed feeding [6]. Even in the early years of the national PMTCT programme, a study done from 2002 to 2003 showed poor infant feeding practices among both women with and without HIV, with less than 10% exclusively breastfeeding at 12 weeks [7]. High rates of formula feeding were also reported among women with HIV, when free infant formula was available [8–10]. In a study done in Cape Town, over 90% of postpartum women with HIV reported ever using infant formula, including a large proportion of women who had also breastfed [8]. Also, in a large, national facility-based evaluation conducted in 2010, almost two-thirds of HIV exposed infants aged four to eight weeks were reported to have received infant formula and no breast milk [9].

Between 2010 and 2015, there have been several changes in infant feeding and PMTCT guidelines in South Africa, with the withdrawal of free infant formula, provision of extended infant and maternal ARV prophylaxis for breastfeeding women, and the most recent change in 2015 being the adoption of Option B+, lifelong antiretroviral therapy (ART) for all pregnant and postpartum women with HIV, regardless of the level of immune suppression or infant feeding mode [4, 5, 11]. Most of the available literature on infant feeding practices in South Africa predates implementation of these policy changes, and tends to focus on women with HIV. Hence, we conducted a cross-sectional survey in Johannesburg, South Africa, to evaluate infant feeding knowledge, perceptions, and practices among both women with and without HIV.

## Methods

### Study setting and participants

A survey was undertaken from April 2014 to March 2015 in Soweto, Johannesburg. Ten medium and high volume public healthcare facilities, with 50–100 first antenatal care visit patients per month, and providing both antepartum and postpartum care, were selected for the study. Five facilities were selected from each of the two sub-districts in Soweto, a densely populated low and middle income area. At the time of study, the HIV prevalence among pregnant women attending antenatal clinics in Soweto was approximately 29% (unpublished programme data). All pregnant women receive group counselling on infant feeding, with individual counselling also provided for women with HIV. Group and individual counselling on safe infant feeding practices is provided by trained lay-counsellors and professional nurses, and at the time of the study reflected the 2013 and 2015 South African PMTCT guidelines, which are adapted from the WHO guidelines on infant feeding in the context of HIV infection [1, 5, 11]. In both the 2013 and 2015 South African guidelines, the recommendation is for women with HIV to exclusively breastfeed for six months, with introduction of complementary food from six months and continued breastfeeding for up to 12 months [5, 11]. In the 2013 guidelines, there was provision for either extended infant or maternal antiretroviral prophylaxis for the duration of breastfeeding in women who did not need lifelong ART for their own health [11]. The current recommendation, based on the 2015 guidelines, is for all pregnant and breastfeeding women with HIV to be initiated on lifelong ART, regardless of level of immune suppression [5]. Women without HIV are counselled to exclusively breastfeed for six months, with introduction of complementary food from six months and continued breastfeeding for up to two years or more [5].

A systematic sampling procedure was used to recruit women with and without HIV during pregnancy and up to 12 months postpartum. Every fifth pregnant or postpartum client to enter the facility was invited to participate in the study, and if she declined the next client was recruited. Pregnant women were recruited if they were attending a follow-up antenatal visit, and postpartum if they were attending a clinic visit beyond the sixweeks visit. The study was approved by the University of the Witwatersrand Human Research Ethics Committee (No. M130801), and access to the facilities was granted by the Johannesburg District Health Department.

### Data collection

Women who agreed to be part of the study, and were 18 years and older, were interviewed using a semi-structured questionnaire. All study participants provided written informed consent. Approximately 20 antepartum and 20 postpartum women were interviewed in each facility. The questionnaire was in English and the interviews were conducted by lay-educators who had a good understanding of the infant feeding guidelines as they provided PMTCT education sessions at the sites. The interviewers completed the questionnaire and were trained on how to explain the technical terms, and where necessary, translated from the English interview into the two predominant vernacular languages in Soweto, Sesotho and Zulu. A field manager and the study investigators provided supervision throughout the study period.

The questionnaire had subsections in the following order: demographics, self-reported HIV status and ART use if HIV-infected, infant feeding intentions among pregnant women and practices among postpartum women, and factors influencing the intentions and practices, sources of infant feeding information, general knowledge on infant feeding, perceptions on exclusive breastfeeding, knowledge of infant feeding in the context of HIV infection, and factors supportive of exclusive breastfeeding. The questions in the questionnaire were a mixture of open-ended and closed questions. The closed questions had predefined options of Yes/No, True/False, and multiple response items. The Food and Agriculture Organization of the United Nations (FAO) guidelines for assessing nutrition-related knowledge, attitudes and practices manual lists open-ended and closed questions as types of questions that can be used in infant feeding surveys [12].

In formulating the knowledge questions, we identified key items that are integral to core knowledge on safe infant feeding practices in general and in the context of HIV infection. The questions were based on the WHO definitions and guidelines on infant feeding, South African PMTCT guidelines and published literature [1, 5, 11, 13, 14]. Several studies on infant feeding, conducted in sub-Saharan Africa, have used questions based on WHO definitions and guidelines on infant feeding to assess knowledge in pregnant and postpartum women with and without HIV [7, 15–19]. In our survey, a total of 16 key-knowledge questions were selected for both pregnant and postpartum women (Table 1). Questions on general knowledge of safe infant feeding practices were asked first, followed by questions on infant feeding in the context of HIV infection, and the questions were ordered such that earlier questions in the questionnaire did not influence responses to later questions. Each question was assigned a score of 1 for a correct answer, and 0 for an incorrect answer, with a maximum score of 16. To assess perceptions on exclusive breastfeeding, we formulated four statements with response options of True, False and Don’t know. The four statements were on perceived ease of exclusive breastfeeding and factors that impact on this practice. Our statements to assess perceptions on exclusive breastfeeding are similar to those used by Kafulafula et al. and Mogre et al. in assessing attitudes towards exclusive breastfeeding [19, 20].

**Table 1:** Questions on safe infant feeding practices

General knowledge questions on safe infant feeding practices 1. If one chooses to exclusively breastfeed, how long should it be for? 2. If one chooses to exclusively formula feed, how long should it be for? 3. If exclusive breastfeeding is the option chosen, what else can be given to the baby? 4. If exclusive formula feeding is the option chosen, what else can be given to the baby? 5. When an infant cries it always means that it’s hungry. 6. Breast milk is better than formula milk. 7. Formula milk is almost as good as breast milk. 8. Infants who are breastfed are less likely to get infections. 9. All mothers should breastfeed for up to two years.
Questions on safe infant feeding practices in the context of HIV 1. Does the HIV-infected mother who is breastfeeding need to take any medication? 2. If she needs to take medication, what does she need to take? 3. If she takes medication, how long does she need to take it for? 4. Does the baby need to take medication during breastfeeding? 5. If the baby needs to take medication, what does it need to take? 6. If the baby takes medication, how long does it need to take it for? 7. With the HIV-infected mother, when is the infant tested again after stopping breastfeeding?

### Data analysis

Study data were collected and managed using REDCap electronic data capture tools hosted at the University of the Witwatersrand, and analysed using Dell Statistica (data analysis software system), version 12, software.dell.com, Dell Inc. (2015) [21]. Means, with standard deviations (SD), and medians with interquartile ranges (IQR) were calculated for continuous data, and frequencies were determined for categorical data. Student’s t-tests and Mann-Whitney tests were used for comparison of means and medians, respectively. Chi-squared and Fisher’s exact tests were used for categorical data and statistical significance was accepted as the two-tailed *p* < 0.05. Multiple logistic regression analyses were done to determine associations between independent variables and the binary outcome of intention to exclusively breastfeed among pregnant women and exclusive breastfeeding among postpartum women with infants less than six months of age. Variables that had an association with the binary outcomes with a *p* - value of 0.20 or less in the bivariate analyses were included in the final multivariate model. Variables of particular interest in this study, such as HIV status and feeding knowledge scores, were also included in the final models, irrespective of their significance in the bivariate analyses. The Wald test statistic was used to assess the significance of association between the independent variables, and the intention and practice of exclusive breastfeeding.

## Results

A total of 190 pregnant and 180 postpartum women met the inclusion criteria and were included in the final analysis. The demographic and socio-economic characteristics of study participants are presented in Table 2. Among pregnant women, 38.9% (74/190) were HIV positive, and 65/74 (87.8%) had disclosed their HIV status, 54/65 (83.1%) to their partner. The mean age of pregnant women with HIV was 29.3 ± 6.6 years, and 15.1% (11/73) were primigravida. Pregnant women without HIV were younger with a mean age of 27.0 ± 6.2 years (*p* = 0.019), and a greater proportion, 26.4% (29/110), were primigravida (*p* = 0.070). At the time of interview, the mean number of antenatal clinic visits attended was similar for both groups of pregnant women, 3.1 ± 1.0 for women with HIV and 3.0 ± 1.1 for women without HIV, *p* = 0.375. Among postpartum women, 67/180 (37.2%) were HIV positive, and 61 (91.0%) disclosed their HIV status, 82.0% (50/61) to their partner. Postpartum women without HIV were a mean 2.1 years younger than postpartum women with HIV, *p* = 0.042.

**Table 2:** Characteristics of pregnant (*n* = 190) and postpartum women (*n* = 180)

	Pregnant women			Postpartum women		
	HIV infected	HIV uninfected	*p*-value	HIV infected	HIV uninfected	*p*-value
Socio-demographic variables						
Age in years, mean (SD)	29.3 (6.6)	27.0 (6.2)	0.019	29.7 (6.0)	27.6 (6.8)	0.042
Employed, *n* (%)	29/74 (39.2)	35/115 (30.4)	0.214	19/67 (28.4)	34/113 (30.1)	0.806
Level of education, *n* (%)						
≤ 11 years of school	37/74 (50.0)	33/116 (28.4)	0.006	34/67 (50.7)	37/113 (32.7)	0.009
Completed school	29/74 (39.2)	57/116 (49.1)		30/67 (44.8)	56/113 (49.5)	
Tertiary education	8/74 (10.8)	26/116 (22.4)		3/67 (4.5)	20/113 (17.7)	
House with electricity and water, *n* (%)	64/74 (86.5)	108/116 (93.1)	0.129	54/67 (81.8)	99/113 (87.6)	0.289
Living with parents, *n* (%)	17/72 (23.6)	44/112 (39.3)	0.028	24/67 (35.8)	45/112 (40.2)	0.562
Obstetric history						
Gestational age in weeks, mean (SD)	27.2 (8.9)	27.4 (7.8)	0.857	–	–	–
Infant age in months, mean (SD)	–	–	–	5.9 (3.0)	5.5 (3.0)	0.408
Number of previous pregnancies, median (IQR)	2 (1–2)	1 (0–2)	0.028	2 (1–3)	2 (1–2)	0.018
Ever breastfed, *n* (%)	42/74 (56.8)	61/116 (52.6)	0.573	49/67 (73.1)	58/110 (52.7)	0.007
Pre-pregnancy HIV diagnosis, *n* (%)	28/74 (37.8)	–	–	22/67 (32.8)	–	–
On ART, *n* (%)	69/74 (93.2)	–	–	58/66 (87.9)	–	–

### Infant feeding knowledge, intentions and perception among pregnant women

Pregnant women with HIV had better overall knowledge on safe infant feeding practices, scoring a mean two points higher than pregnant women without HIV, *p* < 0.001 (Table 3). This difference related both to better knowledge about infant feeding in the context of HIV and to general infant feeding practices. Women without HIV scored poorly on questions relating to definition and duration of exclusive breastfeeding and formula feeding, and also on questions related to antiretroviral prophylaxis for the HIV exposed infant.

**Table 3:** Infant feeding knowledge among pregnant (*n* = 190) and postpartum (*n* = 180) women

	Pregnant women			Postpartum women		
	HIV infected (*n* = 74)	HIV uninfected (*n* = 116)	*p*-value	HIV infected (*n* = 67)	HIV uninfected (*n* = 113)	*p*-value
^a^Mean score (SD)						
General infant feeding knowledge	7.0 (1.6)	6.1 (2.2)	0.001	6.4 (2.1)	6.5 (2.1)	0.639
HIV-related infant feeding knowledge	5.5 (1.2)	4.4 (1.7)	<0.001	5.7 (1.0)	4.0 (1.6)	<0.001
Overall knowledge	12.5 (2.3)	10.4 (3.3)	<0.001	12.1 (2.5)	10.6 (2.9)	0.001

^a^General knowledge scores are out of a maximum of 9, HIV-related knowledge out of 7 and overall knowledge out of 16

A greater percentage of women without HIV, 80.9% (93/115), reported that they intended to exclusively breastfeed, compared to 64.9% (48/74) of pregnant women with HIV, *p* = 0.014 (Table 4). In the multiple logistic regression analysis (Table 5), prior breastfeeding experience and higher knowledge scores on safe infant feeding practices related to HIV were positively associated with a reported intention to exclusively breastfeed. HIV infection, older maternal age, and advanced gestation at the time of interview were all negatively associated with an intention to exclusively breastfeed. Not having HIV was positively associated with an intention to breastfeed, adjusted odds ratio (AOR) 3.60, 95% CI 1.50, 8.62, *p* = 0.004. Although not statistically significant, higher levels of education and having discussed infant feeding intentions were negatively associated with intended exclusive breastfeeding. There was no significant association between marital status, employment, living with parents and a reported intention to exclusively breastfeed.

**Table 4:** Infant feeding intentions among pregnant women, and feeding practices in postpartum women

Infant feeding intentions (Antepartum)	HIV infected *n* (%)	HIV uninfected *n* (%)	*p* - value
Intention to exclusively breastfeed	48/74 (64.9)	93/115 (80.9)	0.014
Discussed infant feeding intention	67/74 (90.5)	105/116 (90.5)	0.995
With partner	55/67 (82.1)	83/105 (79.0)	0.676
With family	20/67 (29.9)	39/105 (37.1)	0.338
Infant feeding practices (Postpartum)	HIV-infected *n* (%)	HIV-uninfected *n* (%)	*p*-value
Exclusive breastfeeding, infants < 6 months	23/36 (63.9)	45/64 (70.3)	0.509
Any breastfeeding, infants ≥ 6 months	14/31 (45.2)	28/49 (57.1)	0.296
Discussed infant feeding decision	55/67 (82.1)	101/112 (90.2)	0.118
With partner	39/55 (70.9)	80/101 (79.2)	0.085
With family	25/55 (45.5)	41/101 (40.6)	0.890

**Table 5:** Multivariable logistic regression analysis of factors associated with intention to exclusively breastfeed among pregnant women

Factors	Adjusted odds ratios	95% confidence intervals	*p*-value
Maternal age (per year)	0.93	0.87, 0.99	0.033
Gestational age (per week)	0.94	0.90, 0.99	0.015
HIV-uninfected	3.60	1.50, 8.62	0.004
≥ 12 years of school	0.47	0.20, 1.07	0.070
Breastfeeding in previous pregnancy	3.86	1.61, 9.23	0.002
Discussed feeding intentions with someone	0.14	0.02, 1.16	0.068
General knowledge score (per unit increase)	1.04	0.84, 1.28	0.719
HIV-related knowledge score (per unit increase)	1.23	0.93, 1.63	0.004

Chi-square = 33.65; *R* ^2^ = 0.159; Log likelihood ratio = – 88.90

The majority of women reported that their infant feeding choice was based on information received from attending antenatal clinics, 83.6% (*n* = 73) and 73.3% (*n* = 116) for women with and without HIV, respectively. However, 27 of the 74 women with HIV (36.5%), including 12 (44.4%) diagnosed during the index pregnancy, were influenced by their HIV status in deciding on an infant feeding option. Of these 27 women, 19 (70.4%) elected to formula feed, five (18.5%) reported that they would breastfeed for six months or less because of fear of infecting their infants, and three (11.1%) were undecided about what infant feeding option to choose.

The perception among both groups of pregnant women was that enough information was given in healthcare facilities to encourage mothers to exclusively breastfeed, 87.8% (65/74) among women with HIV and 86.8% (99/114) among women without HIV. However, the majority of pregnant women thought it was difficult to exclusively breastfeed and that most mothers do not practice exclusive breastfeeding (Table 7). Cultural factors and influence from elders in the family were perceived to be barriers to exclusive breastfeeding in both groups. About a third of both groups of pregnant women, 32.9% (24/73) of women with HIV and 35.3% (41/116) of women without HIV, thought that if a mother elects to formula feed, people will think that she has HIV.

### Infant feeding knowledge, practices and perceptions among postpartum women

Differences were observed in overall knowledge between postpartum women with and without HIV (Table 3). Women with HIV had a mean 1.7 higher score on knowledge of safe infant feeding practices in the context of HIV infection, while the knowledge scores on general infant feeding were similar for both groups.

Among postpartum women, 53.7% (36/67) of women with HIV and 56.6% (64/113) of women without HIV had infants less than six months of age. The mean age of the infants less than six months was 3.6 ± 1.3 months and 3.2 ± 1.3 months for women with and without HIV, respectively, *p* = 0.160. There was no difference in the proportion that reported exclusive breastfeeding – 23/36 (63.9%) among women with HIV and 45/64 (70.3%) among women without HIV, *p* = 0.509 (Table 4). A total of 6/36 (16.7%) women with HIV reported that they were exclusively formula feeding, and 7/36 (19.4%) that they were mixed feeding. The results of the multiple regression analysis of factors associated with exclusive breastfeeding in postpartum women with infants less than six months of age are presented in Table 6. Higher scores on general knowledge of safe infant feeding practices were positively associated with exclusive breastfeeding, AOR 2.18, 95% CI 1.52, 3.12, *p* < 0.001. Although not statistically significant, having HIV was positively associated with exclusive breastfeeding. Women who were employed, single and had older infants had significantly lower odds of exclusive breastfeeding. There was no association between exclusive breastfeeding and level of education or living with parents among postpartum women.

**Table 6:** Multivariable logistic regression analysis of factors associated with exclusive breastfeeding among postpartum women

Factors	Adjusted odds ratios	95% confidence intervals	*p*-value
Maternal age (per year)	1.09	0.98, 1.21	0.098
Infant age (per month)	0.59	0.38, 0.94	0.025
HIV-infected	1.94	0.44, 8.62	0.382
Not married or cohabiting	0.19	0.05, 0.77	0.020
Employed	0.08	0.02, 0.35	0.001
General knowledge score (per unit increase)	2.18	1.52, 3.12	< 0.001
HIV-related knowledge score (per unit increase)	0.74	0.50, 1.10	0.135

Chi-square = 50.40; *R* ^2^ = 0.402; Log likelihood ratio = – 37.48

For the majority of postpartum women, 72.2% (130/180), reported that the main source of information on infant feeding was that from healthcare facilities. Perceptions expressed on factors relating to exclusive breastfeeding were similar to those of pregnant women (Table 7). Of postpartum women with HIV, 40.1% (26/65) thought that women who elect to formula feed would be thought of as having HIV, compared to 27.9% (31/111) of women without HIV, *p* = 0.099.

**Table 7:** Perceptions on exclusive breastfeeding

	Antepartum		Postpartum	
	HIV infected *n* (%)	HIV uninfected *n* (%)	HIV infected *n* (%)	HIV uninfected *n* (%)
It is easy to exclusively breastfeed for six months	26/74 (35.1)	36/116 (31.0)	29/67 (43.3)	45/113 (39.8)
Most mothers exclusively breastfeed for six months	21/72 (29.2)	31/116 (26.7)	23/67 (34.3)	49/113 (43.4)
Cultural factors prevent most mothers from exclusively breastfeeding	55/73 (75.3)	70/116 (60.3)	42/67 (62.7)	73/113 (64.6)
Influence from elders prevent most mothers from exclusively breastfeeding	61/73 (83.6)	89/116 (76.7)	50/67 (74.6)	81/113 (71.7)

## Discussion

Findings from our study suggest that infant feeding patterns among women with HIV have changed, following changes in infant feeding guidelines. A greater proportion of postpartum women with HIV, than reported previously in the South African literature, reported exclusive breastfeeding, and the majority of pregnant women with HIV reported an intention to breastfeed [6]. HIV infection was, however, still a significant factor associated with breastfeeding intentions. Pregnant women who expressed an intention to exclusively breastfeed, compared with other feeding methods, had 3.6 times increased adjusted odds of not having HIV. For all groups of women in the study, healthcare facilities were reported to be the main source of information on infant feeding, and influenced feeding intentions and practices. There were however important knowledge gaps on safe infant feeding practices among both pregnant and postpartum women. Women with HIV had higher knowledge scores than women without HIV, particularly for infant feeding in the context of HIV infection. The perception expressed by most women in the study was that it was not easy to exclusively breastfeed for six months, and that cultural factors and influence from elders in families prevented most mothers from exclusively breastfeeding. There was also a perception in about a third of the participants; those women who elect to formula feed will be perceived as having HIV. This perception on the association of formula feeding with having HIV has been reported previously, and was common when infant formula was available for free in South Africa, for women with HIV who elected to formula feed [22, 23]. Despite this perception, a number of pregnant women with HIV in our study reported an intension to formula feed because of fears of infecting their infants.

We found high rates of reported exclusive breastfeeding among women with and without HIV. In a study done in Malawi, Kafulafula et al. [20] found higher parity, previous positive experience with breastfeeding and positive beliefs about exclusive breastfeeding all associated with a greater intention to exclusively breastfeed among women with HIV. In our study, similar to findings from the Malawian study, prior breastfeeding experience was associated with an intention to exclusively breastfeed. Although not significant, higher knowledge scores on safe infant feeding practices were also linked to a greater probability of an intention for breastfeed. Having HIV infection was associated with decreased adjusted odds of an intention to breastfeed, with a proportion of women with HIV reporting an intention to formula feed because of fears of infecting their infants. Similar to other studies, higher levels of maternal education and discussing infant feeding intentions with others were associated with decreased adjusted odds of an intention to exclusively breastfeed [8, 20]. Zulliger et al. [8] found that women with HIV, with lower levels of education, were more likely to express an intention to breastfeed. Women with higher levels of education are likely to be employed and hence be able to afford infant formula [20]. Disclosure of HIV status has been associated with decreased intended duration of breastfeeding among women with HIV, perhaps because women fear disapproval of extended breastfeeding with the risks of HIV transmission, but this was not significant in our study [20].

Consistent with our study findings, greater knowledge of exclusive breastfeeding has been associated with increased likelihood of exclusive breastfeeding, while maternal employment and infant age have been associated with a decreased practice of exclusive breastfeeding [19, 22]. Studies have shown that women who are employed are more likely not to breastfeed or wean early, and that this is related to the need to return to work after giving birth, the demands of being employed and also the practicalities of breastfeeding while at work [8, 24, 25]. Younger infants are likely to be exclusively breastfed, and this could be related to the perception that breast milk only is inadequate for the older infant [19]. Contrary to the finding on intention to breastfeed, HIV infection was positively associated with exclusive breastfeeding among postpartum women, but the association was weak. A South African cohort study also found that women with HIV were more likely to exclusively breastfeed than women without HIV [7]. This could be a reflection of the misconception that only women with HIV need to practice exclusive breastfeeding, to prevent HIV transmission, and is optional for women without HIV [26]. In most sub-Saharan countries, including South Africa, mixed feeding is the norm and adherence to exclusive breastfeeding raises suspicions of HIV infection, and to avoid the stigma, women with and without HIV may elect to ‘mix feed’ [26–28]. This is somewhat a contradiction given that, as discussed earlier, there is a perception that formula feeding is also associated with having HIV [22, 23]. Availability of male partner support is one of the factors found to mitigate the stigma and encourage exclusive breastfeeding in women with and without HIV [24]. Consistent with this finding, we found not being married and not cohabiting associated with decreased adjusted odds of exclusive breastfeeding. Several other factors contribute to suboptimal infant feeding practices, and include previous experience with infant feeding; perceived inadequacy of exclusive breastfeeding; family and cultural influences; and a need to return to work [8, 24, 27, 29]. Poor knowledge in mothers, as well as inconsistent and inadequate infant feeding counselling by healthcare workers also contribute to unsafe infant feeding practices [8, 24, 27, 29].

It is well-established that mixed feeding is associated with a high risk of MTCT in the absence of either infant or maternal ARV prophylaxis [1]. While maternal ART is protective of breast feeding transmission, adherence to treatment throughout the breastfeeding period is essential for prevention of HIV transmission [30]. In a study done in Zambia, where women received continuous ART starting in pregnancy and throughout breastfeeding, the majority of cases of MTCT occurred in infants older than six months, and adherence to maternal ART was suboptimal in all the cases [30].

Healthcare workers remain the main and important source of information on infant feeding, as we found in our study [27, 31, 32]. The messages need to be consistent as rapidly changing guidelines on infant feeding in the context of HIV infection have led to confusion among both healthcare providers and women accessing care [27, 31, 33]. Data from an established Option B+ PMTCT programme in Malawi however highlighted that most infant feeding counselling still occurs during pregnancy and delivery, with minimal counselling during the postpartum period [32]. Our finding of better knowledge among women with HIV is consistent with findings from other studies as there has been a greater emphasis on counselling and educating women with HIV on safe infant feeding practices and exclusive breastfeeding [26]. A perception has been created that women without HIV do not have to adhere to safe feeding practices, and that exclusive breastfeeding is a recommendation only for women with HIV, for prevention of HIV transmission [26]. This is of concern, as poor infant feeding practices are associated with increased infant morbidity and mortality, independent of HIV infection [24, 34]. Hence safe infant feeding messaging needs to target both women with and without HIV, and continue throughout pregnancy and the postpartum period.

Reported disclosure rates, especially to partners, were high among pregnant and postpartum women with HIV in our study. Disclosure of HIV status to partners, and to family members in those who live in extended families, is associated with adherence to PMTCT interventions, including safe infant feeding practices [27, 33]. Cultural factors and influence from elders have been identified as important factors that impact negatively on breastfeeding practices, consistent with perceptions expressed by the majority of participants in our study [35]. Cultural barriers to exclusive breastfeeding need to be addressed and the interventions must be specific to the local context [35].

There are several limitations to our study. It would have been more desirable to do a longitudinal study to compare intentions and actual postpartum practices in the same group of women, to determine how intentions translate to practice. The study design used was the most efficient use of available resources, and there are several challenges with mobility and loss to follow-up among postpartum women [28]. There could also have been a social desirability bias in reporting of infant feeding intentions and practices, and because the study was quantitative, we did not explore in detail the motivation behind the intentions and practices. Also, undertaking the interviews in the healthcare facilities might have been perceived as a test of the women’s uptake of information given in the facilities rather than their true intentions and practices. Social desirability could have also influenced perceptions expressed by the study participants. The other limitation is that the questionnaire was not translated into the local vernacular languages, but steps were undertaken to minimise any measurement bias by training the interviewers and standardising the translation of technical terms. While the questionnaire used in our survey was not formally evaluated for validity and reliability, similar questions to assess knowledge and perceptions have been used in several infant feeding studies done sub-Saharan Africa, as discussed in the methods section. The HIV prevalence among participants in our study was higher than that reported in the PMTCT programme data and in national surveys, and hence there may have been an overrepresentation of women with HIV. It is likely that women with HIV visit healthcare facilities more frequently for follow-up and to access treatment.

## Conclusions

There is evidence of positive changes in infant feeding patterns, especially among women with HIV, in line with the changing infant feeding guidelines. While the study cannot be generalised to the wider South African population, our findings highlight important issues that still need to be addressed to promote safe infant feeding knowledge and practices among both women with and without HIV. The most important one is to ensure accurate and consistent messaging on safe infant feeding practices for all women during the antepartum and postpartum periods. There is also a need to address cultural factors that are barriers to optimal feeding practices.

## Abbreviations

ART: Antiretroviral therapy
ARV: Antiretrovirals
PMTCT: Prevention of mother-to-child transmission of HIV
WHO: World Health Organization
